# Making Memories: Why Time Matters

**DOI:** 10.3389/fnhum.2018.00400

**Published:** 2018-10-16

**Authors:** Paul Kelley, M. D. R. Evans, Jonathan Kelley

**Affiliations:** ^1^Sleep, Circadian and Memory Neuroscience, The Open University, Milton Keynes, United Kingdom; ^2^Sociology and Applied Statistics Program, University of Nevada, Reno, Reno, NV, United States; ^3^Sociology, University of Nevada, Reno, Reno, NV, United States

**Keywords:** Circadian timing, long-term memory, social time, spaced learning

## Abstract

In the last decade advances in human neuroscience have identified the critical importance of time in creating long-term memories. Circadian neuroscience has established biological time functions via cellular clocks regulated by photosensitive retinal ganglion cells and the suprachiasmatic nuclei. Individuals have different circadian clocks depending on their chronotypes that vary with genetic, age, and sex. In contrast, social time is determined by time zones, daylight savings time, and education and employment hours. Social time and circadian time differences can lead to circadian desynchronization, sleep deprivation, health problems, and poor cognitive performance. Synchronizing social time to circadian biology leads to better health and learning, as demonstrated in adolescent education. In-day making memories of complex bodies of structured information in education is organized in social time and uses many different learning techniques. Research in the neuroscience of long-term memory (LTM) has demonstrated in-day time spaced learning patterns of three repetitions of information separated by two rest periods are effective in making memories in mammals and humans. This time pattern is based on the intracellular processes required in synaptic plasticity. Circadian desynchronization, sleep deprivation, and memory consolidation in sleep are less well-understood, though there has been considerable progress in neuroscience research in the last decade. The interplay of circadian, in-day and sleep neuroscience research are creating an understanding of making memories in the first 24-h that has already led to interventions that can improve health and learning.

Circadian cycles create the patterns of wake and sleep in which short and long-term memories are created. Social factors leading to mistiming wake and disrupting sleep can have a negative impact on these memory functions. Memory processes in the initial 24-h cycle are critical in determining what is encoded into long-term memory (LTM) rather than left to fade in short term memory (STM). There are many stimuli that can trigger LTM but, in this review, we focus on spaced repetition leading to LTM of bodies of complex information, an essential process in learning.

The fundamental role of circadian timing processes has been emphasized by the award of the Nobel Prize in 2017 in physiology or medicine to Hall, Rosbash, and Young for their discoveries of the genetic mechanisms that create circadian 24-h timing in drosophila and humans alike (Bargiello et al., 1984; Siwicki et al., 1988; Hardin et al., 1990; Liu et al., 1992; Price et al., 1998). Circadian 24-h cellular mechanisms are genetic, evolutionarily conserved, and found across all photosensitive forms of life (Bass and Lazar, 2016). In the last 10 years circadian timing has been shown to have a direct impact on human disease, immunity, metabolism, and neurodegeneration (Man et al., 2016; Musiek and Holtzman, 2016; Panda, 2016; Turek, 2016).

Circadian timing can also have a direct impact on human LTM and learning. Circadian clocks are driven by complex genetic and neuroscientific processes. In turn these clocks co-ordinate most 24-h activity including physiological, metabolic, organ and brain functions, including wake/sleep and cognition. When circadian timing is disrupted, then the biological clocks in different tissues can become uncoupled, resulting in a state of internal desynchrony that can have negative impacts on memory creation and health (Foster et al., 2013). Here we consider the central role of modern societies in creating desynchrony when social and biological times are in conflict (Merton, 1936; Sorokin and Merton, 1937; Zerubavel, 1982, 1985; Foster et al., 2013).

There are many ways in which LTM can be created, though it is only recently that the neuron's intracellular synaptic plasticity mechanisms have been discovered in mice (Fields et al., 2005, Fields, 2005). In Fields' research these mechanisms required a “time code” of periods without stimuli to encode LTM. Specifically, the timing pattern was three stimuli separated by two 10-min periods without stimuli. Later, other researchers applied this timing pattern to humans learning complex bodies of structured information (Kelley and Whatson, 2013).

The first section explores recent advances in our understanding of circadian neuroscience, chronotypes, and sleep deprivation. These advances identify research-based applications for improving learning (and health) in adolescent education through synchronizing education times to circadian time. The second section focuses on advances in LTM neuroscience, and an application for improving learning based on these advances leading to creating LTM in humans of complex bodies of structured information. Both circadian and LTM neuroscience are then considered in memory consolidation in sleep.

## Circadian neuroscience

### Biological and social time

Like most life on earth, our physiology, health and behaviors have 24-h rhythms that follow the increasing and decreasing light levels throughout the day and night. These changes in light are the most significant environmental time system and have led to a variety of biological systems to attune animals to circadian timing. In mammals, circadian timing is in effect a two-stage process, with cells individually generating a 24-h rhythm and a master circadian pacemaker establishing more consistent timing throughout the body. In humans the 24-h pacemaker is the suprachiasmatic nuclei (SCN) in the hypothalamus, and the SCN itself is synchronized, or “entrained,” to the variations in light, specifically irradiance. The specific biological mechanism for sensing these changes are triggered when blue light of ~480 nm wavelength enters the eye and strikes photosensitive retinal ganglion cells (pRGC) (Foster and Hankins, 2007). Generally, humans respond most strongly to light levels in twilight (before dawn or after sunset) that entrain circadian timing to the 24-h day.

This pRGC/SCN timing mechanism was discovered in mammals only recently and has implications for human functions including making memories. As circadian time systems adjust physiology and behavior in preparation for expected requirements during the day-night cycle, synchronization to changes in light are critical. Even sensing a single photon of light can be important: the absorption of a ~480 nm wavelength photon by 11-cis-retinaldehyde and the photoisomerization of this molecule in a pRGC leads to a strong signal being sent from the eye directly to the SCN (Wald, 1968; Foster and Hankins, 2007). The number of pRGC cells in the eye is relatively low, and consequently our sense of time depends on the gross amount of light of the appropriate wave length (Lucas et al., 2014). Changing levels of illuminance directly regulate pRGC activity and thus the SCN, creating an automatic, unconscious circadian time system. Indeed, totally blind subjects can retain normal circadian time function, demonstrating the separation of vision and time functions in the human eye (Zaidi et al., 2007).

Socially created lighting environments can also negatively impact on making memories as light itself has an important role in securing optimal physical and cognitive performance (Cajochen, 2007). Light has a direct impact on important aspects of creating memory: light levels act as a neurophysiological stimulant that increases alertness, attention, and improves reaction times. The direct response to bright light has differing impacts depending on the time as has been demonstrated in several ingenious studies (Cajochen et al., 2000; Phipps-Nelson et al., 2003; Rüger et al., 2006). While sunlight and very high light levels can boost human alertness, attention, cognition, and performance, low light levels can have the reverse effect (Lockley and Gooley, 2006; Lockley et al., 2006). For example, Rüger and colleagues concluded light levels may have a wide range of applications, ranging from optimizing a work environment to treating depressed patients. In contrast, many work environments rely on artificial light, reducing light levels far below the lux level of natural light, lowering light-induced alertness, and decreasing access to light with appropriately timed changes in illuminance that are critical in the pRGC/SCN timing system. The overall impact in schools, universities and employment is creating a more uniform, lower light experience throughout the day and increasing light at night, and this may have many direct and indirect impacts. In comparison to natural light levels many artificial light environments can negatively impact on natural circadian timing and health (Chang et al., 2015).

Although modern social behaviors, artificial light, and many other factors may cause individuals to become desynchronized from their circadian timing system, our research team found the most significant chronic desynchronization arises from the introduction of socially determined time or universal time (UTC), and fixed times for education, work and other activities. In order to measure desynchronization caused by UTC, our research team developed a new method of measuring time. We reasoned that UTC uses socially determined time zones, daylight savings time, and discounts changes in illuminance within time zones. In contrast, changes in light depend only on geophysical location and the pRGC/SCN timing mechanism, leading us to measure biological time as Geophysical Biological Time (GBT). GBT uses only the environmental cues of changing light in a specific geophysical location to calculate biological time and determining optimal timings for human activities (Bass and Lazar, 2016; Evans et al., 2017). The differences between GBT and UTC can be important, as shown in studies of Time Zone anomalies (Yasseri et al., 2012) and Daylight Savings Time (Coren, 1996).

### Chronotypes, sleep deprivation, and desynchronization

Although the circadian pRGC/SCN timing system entrains individuals to the GBT 24-h day, there are wide variations between different chronotypes in their responses to light. Chronotypes are most often determined through surveys of wake and sleep times, adjusted for working and non-working days. In a long-running series of chronotype questionnaires building upon use of time survey-based research, Roenneberg and his colleagues established very large data sets that identify chronotype's different wake and sleep patterns (Roenneberg et al., 2004, 2007). From this data and other studies, it has emerged $that chronotype varies considerably with extreme early types waking up when extreme late types fall asleep (Roenneberg et al., 2007). Sleep duration and timing also varies with age, sex, and genetic factors (Foster et al., 2013.

An individual's timing system links to circadian rhythms and 24-h changes in behavior, most noticeably in sleep/wake patterns. Other changes are not often visible, yet circadian rhythmicity is found in almost every physiological, metabolic, and behavioral system, for example core body temperature (Lucas et al., 2014). Desynchronization can therefore have real-life implications in human health and performance (Zhang et al., 2014).

Sleep deprivation influences many aspects of health in ways that impact directly and indirectly on memory and learning processes. Since 1964 sleeping <6.5 h each day has been known to be associated with a range of illnesses and higher death rates (Hammond, 1964). This finding has been the subject of decades of research using a range of methodologies (Cappuccio et al., 2010). Although the links between poor sleep, illnesses and other disorders are well-established, solutions have been more difficult to identify. In part this is because of the nature of sleep: it is complex state, involving multiple brain regions, neurotransmitter pathways, and hormones (Foster and Wulff, 2005). The recommended duration of sleep in adulthood is 7–9 h (National Sleep Foundation, 2017), and sleep of <7 h is associated with a range of health risks, poor mood, and potentially mental health problems, as well as poor cognitive function (Czeisler, 2011). The time-in-day of sleep onset is important as well (as night shifts demonstrate): broadly speaking matching individual's biological sleep onset time is optimal, and the greater the divergence from that time the greater sleep disruption. Indeed, sleep disruption has been shown to impact directly on emotional, cognitive and somatic functions that impact directly on health and learning (Foster and Wulff, 2005; Wulff et al., 2011).

Sleep deprivation is clearly associated with poorer cognitive processes (Pilcher and Huffcutt, 1966; Thomas et al., 2000; Van Dongen et al., 2003), and studies have also found poor communication, decreased concentration and cognitive performance, unintended sleeps, decreased motor performance, increased risk taking and changes in mood pattern, specifically depression (Foster and Wulff, 2005: Kelley et al., 2015). These factors directly or indirectly impact negatively on learning and memory processes (Curcio et al., 2006: Diekelmann and Born, 2010; Escribano et al., 2012). Other studies have shown restricted sleep is associated with health issues that may also affect learning and memory including impaired immune response, metabolic disorders, diabetes, hypertension, anxiety, depression, and obesity (Luyster et al., 2012), arising from the neuroscience of sleep loss (Krause et al., 2017).

### Circadian timing and adolescent education: later starts that improve learning and health

The differences between sleep patterns in adolescence and social times for education lead to chronic sleep deprivation. This can be illustrated by considering the school starting time controversy in U.S. middle and high schools. In the U.S. education start times average 08:03 in middle and high schools (Wheaton et al., 2015), and this is too early for older adolescents, leading to poorer health and academic performance (Schmidt et al., 2009; Kirkby et al., 2011; Basch et al., 2014). Changing to later start times in education has been strongly advocated by the Centers for Disease Control and Prevention, the American Academy of Pediatrics, and the American Medical Association (American Academy of Pediatrics, 2014). The specific recommended change is that middle and high schools should open no earlier than 08:30. The recommendation was based on research showing health, mental health and performance were at greater risk when start times were earlier.

The circadian biology of adolescence explains the problems of school timing. Starting with the pioneering of Carskadon (Carskadon et al., 1993; Carskadon and Dement, 2005; Millman, 2005; Hagenauer et al., 2009; Carskadon, 2011), US school times were demonstrated to be desynchronized from adolescent biological timing. It has been suggested that the end of changes in the adolescence tendency to later wake/sleep circadian timing should be used as the marker for the end of puberty (Roenneberg et al., 2004). This proposal was based on evidence that in childhood there is a shift to later wake/ sleep times and females start this process about 1.4 years earlier than males and end this process about 1.4 years earlier, both in line with puberty (but see Sørensen et al., 2012). The genetic basis of these changes by age and sex were confirmed in a range of studies (Duffy et al., 2011; Giedd et al., 2012) many after Roenneberg's proposal. The estimated difference between the mean wake/sleep time of adolescents and the mean of other ages ranges from 2 to 2.7 h in different studies. Such a major difference in wake/ sleep times has been taken as the underlying cause of the mismatch between adolescent wake/sleep times and U.S. school start times. The temporal misalignment between the sleep timing for adolescence and educational institutions' usual start hours causes significant sleep loss. Sleep loss, in turn, impairs academic performance and elevates risks of obesity, depression, and drug abuse. The biological mechanisms through which early starts increase health risks and lower performance are well-established (Carrell et al., 2011; Kirkby et al., 2011). These biological changes in the timing of sleep propensity underlie the conflict with education start times and, just as in sleep-deprived adults, chronic sleep deprivation has negative impacts on cognition (Lockley et al., 2004).

The medical and circadian research evidence for later school starts is overwhelming. The medical community's recommendation that middle and high schools in the U.S. should start no earlier than 08:30 is clearly justified. Schools in many states and other countries such as South Korea are changing to later starts and finding students sleep more, achieve better results and better health. However, moving starting times later than 08:30 does not clarify what wake time is optimal for adolescents of different ages. This issue was addressed by our research team, growing out of our work with Oxford University's Sleep and Circadian Neuroscience Institute. Synchronizing education to adolescent circadian neuroscience evidence led to projections that for adolescents at age 16 starting times within the range 10:00–10:30 were appropriate, whereas starting at age 18 starting times of 11:00–11:30 were appropriate (Kelley et al., 2015). Our research trial was in a UK school that changed its 08:50 start to a 10:00 starting time for 2 years. There were highly significant improvements: absence due to illness fell by 50%, and academic outcomes rose 17% compared to schools nationally. We concluded school starting times that were better aligned with adolescent sleep, chronotype patterns and circadian neuroscience research are practical and beneficial (Kelley et al., 2017). Yet it was becoming clear that desynchrony between circadian and social timing can affect different ages and chronotypes in different ways.

### Circadian and social time synchronization

When there is circadian synchrony, as when social time matches biological time, humans function well. When there is desynchrony it can lead to pathologies and dysfunction, including poorer cognitive function. An important factor in sleep loss is different chronotypes, and the failure to recognize the mismatch between starting times and sleep patterns, and the differing impact on chronotype groups, has limited our understating of the complexities of circadian timing and social time. For example, though the number of evening chronotypes increases with adolescence, there remain morning and in-between chronotypes within the adolescent population. Definitely morning and morning chronotypes suffer far less sleep deprivation with early starts in adolescence, or even have no sleep deprivation caused by school starting times. In contrast, the sleep deprivation for definitely evening chronotypes is far greater and can reach 3 h a day or more. Therefore, circadian chronotype analysis is necessary to more accurately assess the impact on groups and individuals.

These circadian time analyses may help explain the findings that evening chronotypes perform less well in schools, are more likely to have depressive symptoms, and have higher levels of obesity and is consistent across studies of older adolescents (Giannotti et al., 2002; Randler and Frech, 2006; Escribano et al., 2012; Hsu et al., 2012; Preckel et al., 2013). Studies of evening types specifically show these symptoms from relatively early in adolescence through university, and this supports a thesis that evening chronotypes are more likely to be negatively affected by sleep loss than other chronotypes (Kelley et al., 2015). Alternatively, later start hours are associated with longer sleep, better health, and better performance, and most studies conclude that the key variable is sleep loss compared to adequate sleep (Borlase et al., 2013; Wahlstrom et al., 2014).

The conflict between biological and social time is endemic in modern society because of the use of social time systems that are not synchronized to biological time leading to unintended consequences. This may prove difficult to change as social time itself has a historical legacy of industrialization, institutionalization and traditions such as a conventional 9-to-5 working day (Ford, 1926). Although it is not within the scope of this review, societies generally are slow to change, especially in relation to work and life patterns of behavior. To better understand social processes, sociologists have developed a range of analytical tools to track changes and their causes, often working with extremely large data sets. One specific methodology used extensively in sociology is surveys, including the measurement of time. Prior sociological research on survey design showed that during exploratory research very detailed questions about time-related actions and feelings produced much more reliable data than summary questions (Tourangeau et al., 2000; Schaeffer and Presser, 2003). In circadian research large-scale research into circadian patterns of sleep and wake have taken a different survey approach, concentrating just on biological behaviors.

Our research team created a new sociological method to assess chronotype variations in undergraduates using a 24-h time scale of when subjects were at their best. Analysis of the data from 190 undergraduates aged 18–19 was similar to Roenneberg's data suggesting very wide circadian variations indeed (Roenneberg et al., 2007). Using the more detailed time scores meant the new sociological method could track changes in self-assessed alertness throughout wake. The best time for starting using this approach was 11:00 or 12:00. Using the neuroscience-based approach based on local light changes in Reno, Nevada the best time was 11.27 GBT. Projections from our earlier work suggested 11:00 to 11:30. There are two important findings arising from chronotype variations and being at their best measures for each hour: first, any fixed morning start time in the university would lead to some sleep deprivation or disadvantage some chronotype group, and second, no hour in the day for learning would be optimal for all chronotype groups (Evans et al., 2017). The conclusion that much later starting times were required in this age range was expected, but the conclusion that there was *no* single starting time produced optimal times for all chronotypes was not. In a social time frame, small differences between chronotypes' optimal performance are manageable, but very large differences would pose fundamental questions about current social timing. Thus, desynchrony between circadian and social timing can affect different ages and chronotypes in different ways.

Progress in genetic circadian research has also been rapid in the period 2013–2018. An elegant experiment demonstrated sleep loss and adequate sleep in the same subjects, and the genetic mechanisms of sleep loss (Möller-Levet et al., 2013). The 26 female and male participants (mean age 27.5) had a week of sleep restriction of 5.7 h, and a week of adequate sleep of 8.5 h. Controlling for a wide range of variables, whole-blood RNA samples were used in transcriptome analysis, and these showed 711 genes up- or down-regulated by insufficient sleep. These changes demonstrated the negative impacts of sleep deprivation and the positive impact of adequate sleep in the same people (see also Jonsson et al., 2016). Insufficient sleep disrupted circadian regulation of genes and was not sufficient to maintain alertness and performance during wake. This was a very clear demonstration of the importance of sleep and the underlying biological mechanisms associated with a shift from adequate sleep to restricted sleep. Möller-Levet's research can be examined in relation to time patterns in sleep itself, specifically sleep cycles that are repeated during the full duration of sleep. Sleep cycles are about 90 min long, and occur four or five times in a sleep episode (Carskadon and Dement, 2005; Abel et al., 2013). Each sleep cycle is characterized by four sleep stages including rapid eye movement (REM), slow wave sleep and sleep spindles all of which are critical in the consolidation of learning and long-term memories. Restricting subjects to 5.7 h would only allow three complete sleep cycles, and adequate sleep of 8.5 h would allow more than 5 complete sleep cycles. More recently genetic circadian research has demonstrated long-term circadian disruption impacts on human heath (Turek, 2016), immunity (Man et al., 2016), metabolism (Panda, 2016), and neurodegeneration (Musiek and Holtzman, 2016).

Circadian synchrony when social time matches biological time presents many challenges, but the alternative of continuing to allow desynchrony that causes poorer health, mental health and cognitive function is untenable. The surge in home working in many countries including the U.S. enables better, even optimal circadian synchrony, is the most positive example of society adjusting to neuroscientific realities, even if it is unintentional consequence of new technologies. Seemingly intractable issues such as night shifts are amenable to improvement by matching chronotypes to working hours (Vetter et al., 2015), and better night shift and recovery work patterns (Cappuccio et al., 2009). The direct challenge to social times in schools is based on medical, sleep, and circadian evidence, and indicates a significant change in attitudes to social time. In general, the case has been made for circadian synchrony that protects sleep duration, while chronotype variation is less well-understood.

## Long-term memory neuroscience

### LTM and learning complex bodies of structured information

New techniques such as scanning technologies have allowed a better understanding of the neuronal ensembles formed in memory processes. Experimental results suggest learning and memory occur in the context of continual structural synaptic plasticity such as formation and elimination of synapses (Chklovskii et al., 2004; Holtmaat and Svoboda, 2009). Understanding these processes can be enhanced through modeling potential mechanisms to explain experimental findings (Knoblauch and Sommer, 2016; Knoblauch, 2017) or apply them in a new context (Popovych et al., 2015; Abdou et al., 2018). The most significant recent discoveries are in glia biology, and the roles of astrocytes and myelin in cognition and learning (Fields et al., 2014). For example, myelination sheathes regulate the speed and synchrony of synaptic firing, are vital in information processing, links between different cortical regions, and fundamental memory and cognition processes. In contrast, myelin also limits critical periods for learning through inhibiting proteins that reduce synaptogenesis and axon sprouting. Astrocytes impact on synaptic plasticity, long-term potentiation (LTP) in cortex and hippocampus, spike-timing dependent cortical, plasticity, hippocampal long-term depression (LTD) and working memory. In addition, astrocytes can severely restrict LTM formation, restrict LTM consolidation in sleep, and directly influence the SCN during sleep (Brancaccio et al., 2017).

The degree of synchronization of socially determined times to circadian time can impact on learning and memory processes within each 24-h cycle. On waking alertness and performance varies with sleep loss the night before. Wake alertness varies through the day by chronotype and other circadian and biological factors but environmental stimuli are not bound by these predictive biological in-day patterns. When alertness is low stimuli are more likely to trigger short-term memories (STM), and LTM is less frequent unless the stimuli are highly relevant, emotional, novel, repeated, or extreme (Fields, 2005).

LTM processes are the fundamental basis of learning and memory processes and, like the circadian processes, they are biologically determined through evolutionarily-conserved timing mechanisms. Here we focus on cAMP response element-binding protein (CREB) in intracellular LTM functions and timing and the associated CREB-based spaced learning (CBSL) to illustrate biological timing of human LTM processes. The molecular and systems biology of memory has been well-established in broad terms during the last century. Progress has often been slow, with hypotheses that have been proved only after decades of research. Learning is characterized as the biological processes in acquiring information from the environment and consolidation in sleep (Atkinson and Shiffrin, 1968; Kandel et al., 2014). Studies of Aplysia to mammals provide a coherent model of simple neuronal plasticity developing to more complex processes in mammals.

In the early studies of memory, differences between Pavlovian memory encoding in dogs (Pavlov, 2010) and Ebbinghaus's memory retrieval and loss in humans (Ebbinghaus, 1913) emerged. Ebbinghaus's initial findings continue to be researched, notably memory retrieval testing where the intervals between retrieval practices were shown to be important (Cepeda et al., 2008; Karpicke and Roediger, 2008; Karpicke and Bauernschmidt, 2011; Karpicke and Blunt, 2011; Smith et al., 2016), and the similar timing issues in the processes in memory loss (Scoville and Milner, 1957; Gong et al., 2003).

In this review we focus on the initial circadian cycle, and on purposeful memory encoding and consolidation. LTM formation is now understood to depend on Long-Term Potentiation (LTP) and late-long -term potentiation L-LTP. L-LTP depends on protein kinases such as MARK and PKA signaling to CREB and is dependent on gene transcription (Frey et al., 1993; Bourtchuladze et al., 1994; Abel et al., 1997; English and Sweatt, 1997; Perazzona et al., 2004). Repeated but spaced periods of stimuli can initiate intracellular mechanisms activating genes, initiating the production of proteins (Scharf et al., 2002; Morris, 2003; Hernandez and Abel, 2008). These studies show proteins then can strengthen sensitized synapses, triggering LTP, and LTM encoding (Frey and Morris, 1997; Lu et al., 2008; Fields, 2011; Moncada et al., 2011). The discovery of a critical period for these processes had been identified earlier (Nguyen et al., 1994).

Behavioral studies with different species showed that the timing of stimuli was a critical variable in LTM encoding. In a classic study, honeybees were trained to find food locations with 30 s, 3 min, and 10-min spaces with no training stimuli. The honeybees trained with 10 min spaces were the only group that achieved memory encoding and consolidating, reaching almost 100% accuracy on the third day, demonstrating long-term memories had been created (Menzel et al., 2001). In Drosophila spaced learning can produce stabilized LTM and *de novo* protein synthesis CREB-dependent gene transcription (Perazzona et al., 2004). Further Drosophila research demonstrated *de novo* protein synthesis and LTM after spaced learning *in vitro* and *in vivo*, and a gating mechanism for LTM formation (Chen et al., 2012; Plaçais and Preat, 2013). This has led to a further study demonstrating the evolutionary gain in gating LTM encoding, as creating LTP demands high energy input, in contrast with STM (Plaçais and Preat, 2013). The biological processes of LTP within neurons and synaptic plasticity between neurons create LTM through processes termed synaptic tagging and capture, an increasingly robust model of memory formation in mammals, in a process described in a thorough review (Redondo and Morris, 2011). Within neurons, encoding in transient memory traces in synapses (“tagging”) can be followed by a strengthening process of LTP and L-LTP that can become encoded into LTM. Other neural activity processing before, during and after this can interfere with the encoding process. Synaptic tagging and capture has four elements: the expression of synaptic potentiation with the setting of a local synaptic tag; synthesis and distribution of plasticity related proteins (PrPs); capture of proteins by tagged synapses; and stabilization of synaptic strength (Redondo and Morris, 2011). These processes operate in a distinct time window: the initial stimuli and tagging occurring in the first period of 0–30 min; and 30 min to 2 h for PrPs processes including CREB-dependent gene transcription.

### LTM and CBSL

Broadly speaking, LTP and LTM encoding processes occur in relatively short time scales. LTM can be created by extreme stress events (Pitman et al., 2012), and rapid, involuntary memory creation is the norm in humans. Purposeful learning of skills or in education can be effortful and, at times, making memories proves difficult. The solutions must lie in the biology of encoding memories. Studies in mammalian memory processes *in vitro* rat hippocampus cells clarified the importance of neural impulse activity in triggering LTP that had been first identified in studies of nervous system development (Hebb, 1949; Itoh et al., 1995). Later studies used a pattern of three stimuli separated by 10-min spaces that opened voltage-sensitive calcium channels in the cell membrane, activating signaling pathways to the nucleus. The pattern of impulse firing was echoed in the patterns of calcium flashes, and these were visualized using calcium-sensitive dye and scanning laser confocal microscopy to track pulses of calcium influx following stimuli (Fields et al., 2005). These and other related studies demonstrated there was no need for a specific signaling molecule from the membrane to the nucleus, as the spaced pattern of action potential activity led to CREB phosphorylation and DNA synthesis of zif 268, a gene associated with memory (Dudek and Fields, 2002). Parallel studies in the biochemistry of memory over many years have clarified LTP processes further (Baudry et al., 2011).

Studies on living rats showed patterns of action potential activity have a critical role in long-term synaptic change, providing a direct link between *in vitro* studies of protein-dependent LTP and behavioral studies of LTM retention (Shires et al., 2012). These temporal patterns in LTM encoding have recently been linked to specific molecular LTP processes in a time scale of minutes (Naqib et al., 2012), and, in humans, more rapidly in fMRI studies of face recognition (Xue et al., 2011). There were areas of research into LTM processes that appeared to have clear implications in human learning arising from the importance of time patterns in LTM encoding. These time codes were effective in triggering LTP and LTM in mammals, as R. Douglas Fields and others had demonstrated and thus showed the temporal aspects of Ca2+ dynamics linked the stimulus and cellular response.

Although a single neuron can receive signals from thousands of other neurons, it only has one method of sending a message- firing an impulse through the axion or being silent. Fields argued that it follows that genes in neurons are also turned on and off by the patterns of impulse firing. Fields' lab had been investigating nervous system development and plasticity to find how the brain self-organized itself in nervous system development. They tested fetal mice neurons and were able to show that different time patterns controlled different genes (Itoh et al., 1995). Yet there remained another communication issue- having been triggered by a pattern of impulse firing to impact on a gene, how did the signal travel through the neuron itself and give the nucleus the right message?

Information reaches the nucleus by regulating the influx of calcium ions through voltage-sensitive channels in the cell membrane. Intracellular channels in the brain have high levels of calcium ions, CA^+2^ yet inside the neuron levels are 20,000 times lower. When the neurons fire calcium channels open briefly converting the electrical signaling to a chemical time code CREB relays to a neuron's nucleus. Fields' Lab was able to show different temporal patterns regulated different genes: it was not the quantity of calcium entering the neuron but the temporal code it established.

What was the temporal code that triggered LTP and LTM? After many trials, the lab found if the same high-frequency stimulus is applied repeatedly (three times in their experiments) spaced by 10 min without stimuli, the synapse becomes strengthened in LTP. This CREB-based spaced learning (CBSL) code could occur in the first half hour or up to 2 h later as indicated in the synaptic tagging and capture model. As humans need to acquire LTM of large bodies of complex information in education, CBSL attracted my attention. After years of development (Kelley, 2007), CBSL was finally successfully demonstrated with adolescent subjects and national educational tests in 2013. Surprisingly, the study found that in English national tests subjects taught using CBSL learned quicker and performed equally as well subjects taught conventionally (Kelley and Whatson, 2013).

Other researchers used CBSL successfully in different contexts (Vaz, 2014; Garzia et al., 2016; Hägglöf, 2016) leading to support for wider use in education (Churches et al., 2017) and field trials. The billion-euro FuturICT project proposal recommended CBSL as 1 of 10 ways of meeting education's Grand Challenge to enable people to learn at orders of magnitude more effectively (Reynolds, 2010; Johnson et al., 2012; Bocconi et al., 2013). The UK's Open University Institute of Technology and Singapore's National Institute of Education cited CBSL as a promising education innovation to create LTM (Sharples et al., 2016).

A very different application of CBSL suggests potential applications in medical training (Boettcher et al., 2018). In this random controlled study subjects had comparable baseline skills in suture procedures, but subjects that trained via CBSL were superior in terms of improved laparoscopic performance, knot quality, knot score and suture strength. Additionally, CBCL decreased anxiety and impression of challenge compared to controls. The authors concluded CBSL is very suitable for complex motor skill acquisition like laparoscopic suturing and recommended CBSL be used in training courses and surgical programs.

Ironically the key research question in CBSL development and trials had been whether CBSL timing could create any LTM at all. Even the idea of using a specific time code drawn from mammal *in vitro* experiments with humans was considered impossible by many. It is a measure of CBSL research to date that it has raised the possibility that CBSL could be equally effective and more time-efficient. Perhaps more significant, CBSL shows the how detailed neuroscience research in LTM processes can be used to create potentially powerful applications in education and medical practice.

### Memory consolidation in sleep

During wake learning can be manipulated and easily tested, whereas this is not possible in sleep. This stark contrast between wake and sleep processes has meant that consolidation processes in sleep are less well-understood. New findings suggest while wake is a brain state that enables encoding, sleep is a brain state that enables memory consolidation, and models of memory consolidation processes are beginning to emerge in which the role of REM and slow-wave sleep (SWS) in memory is associated with relevant experience to be re-enforced (Rasch and Born, 2013; Sara, 2017). In a ground-breaking *f* MRI study of human adults, it was possible to demonstrate that encoding and retrieval memory processes were anatomically distinct (Gabrieli et al., 1997). This distinction in location arose from testing the differences between familiar and unfamiliar images, demonstrating specialization of mnemonic processes in the human medial temporal lobe memory systems. These findings also demonstrated *f* MRI neuroimaging could be a powerful tool for tracking memory systems. More frequently human studies of consolidation in sleep rely on tests before and after sleep, and these have indicated that novel, reward, intense, repeated, or emotional stimuli are more likely to be encoded and consolidated (Carr et al., 2011; Wilhelm et al., 2011). Some studies indicate specific features, such as negative emotional events are encoded more often that neutral events (Wiesner et al., 2015), and repetition is most effective when there are specific time codes as in CBSL. Consolidation processes in sleep remain only partially understood, and can occur immediately after a single night's sleep, short periods of quiet restfulness during wake (Carr et al., 2011) or after a longer period.

Human and animal studies alike do not yet answer the fundamental question of consolidation: how do sleep processes identify which memories to consolidate? Logically, new memories that have been through the process of LTP to LTM during wake should trigger consolidation, but the biological processes are only just emerging. Following LTM memory consolidation processes appear to include both hippocampal and cortical sharp-wave ripples with sleep spindles, suggesting cortical ripples may form part of the hippocampal-cortical dialogue during NREM sleep and may be crucial to memory consolidation (Khodagholy et al., 2017; Kitamura et al., 2017). It also appears new LTM may be incorporated into a shared neural ensemble or schema (Andersen et al., 1971; Cai et al., 2016). This process may occur during synaptic down-scaling during slow wave sleep (SWS) (Tononi and Cirelli, 2006; Vyazovskiy et al., 2008). Other studies show postsynaptic dendritic spines are formed on different sets of dendritic branches in response to different learning tasks and are protected from being eliminated when multiple tasks are learned and reactivated during subsequent non-rapid eye movement (NREM) sleep (Yang et al., 2014). This in turn suggests consolidation of new memories into shared neural ensembles may occur during SWS during sharp-wave ripple complexes (SPW-Rs) in sleep that replay neural learned sensory information (Sutherland and McNaughton, 2000; Lewis and Durrant, 2011). In contrast disrupting SPW-Rs, and therefore replay, impairs memory retention and memory consolidation (Girardeau et al., 2009; Ego-Stengel and Wilson, 2010).

Yet how can these complex memory consolidation processes be co-ordinated on a cellular level? In an elegant study, it was possible to show that firing antidromic action potentials was probably a key mechanism (Bukalo et al., 2013). Neurons that participate in SPW-Rs are distinguished from non-participating neurons by firing antidromically, inducing synaptic depression throughout the cell. Subsequent synaptic stimulation delivered after antidromic firing triggers a long-lasting increase in synaptic strength. More generally, this process enables the creation of new neuronal ensembles. It is therefore reasonable to hypothesize that memory consolidation itself is the active processes that occur during SWS, enabled by entopic neuronal firing, linking new memories to other neuronal ensembles and possibly enabling transferring memories through SPW-Rs synchronisations. This would be consistent with sleep deprivation or disruption having a negative impact on hippocampus-dependent memory in both humans and rats and indicating a similar impact on memory consolidation itself during sleep (Abel et al., 2013; Brancaccio et al., 2017; Krause et al., 2017). In humans, different brain areas respond differently by circadian rhythmicity and sleep deprivation: in sustained-attention and cognitive tasks cortical responses decreased significantly with sleep deprivation and circadian misalignment while subcortical areas showed variation that primarily followed circadian modulation (Muto et al., 2016). This ground-breaking *f* MRI study suggests that cognitive processes are more liable to poorer performance from circadian and sleep disruption than other brain functions. This may indicate that both sleep deprivation and circadian misalignment of normal sleep periods might have a negative impact both on encoding and consolidation processes. These complex neuroscience issues are by no means fully resolved.

## Conclusion

The first 24 h of a new memory occurs in a circadian temporal frame in which alertness can be different for individuals, dependent on whether the individual wakes sleep deprived or in synchrony with their circadian rhythms. Social time in UTC can be different than GBT, and start times for school and work tend to be early in most societies, and this will disadvantage evening chronotypes. Sleep deprivation can have a negative impact on learning and memory processes, decreasing the ability to encode new memories though LTP and LTM processes, and may disrupt consolidation in sleep. Individuals have distinctive circadian patterns by chronotype that determine the best times for cognitive functions, as shown in optimum times in their 24-h profiles that identify best times (Evans et al., 2017). Studies of learning and memory that do not control for circadian variations may be flawed or fail to identify chronotypes as a significant factor in the variations in outcomes.

Currently rigorous application of discoveries in learning and memory neuroscience to human learning and memory are rare. It may be that CBSL is the first such application using a specific time code of activity and rest and drawn from primary neuroscience research that is able to offer detailed account of learning in the first 24-h period. There is every reason to think that different timing protocols will be identified by human neuroscience research. Although sleep and LTM consolidation are less accessible to experiments, sleep has time patterns of activity and rest every 90 min that can be tracked (Carskadon and Dement, 2005) and there is growing evidence that can clarify the different functions of REM and SWS sleep in consolidation processes (Li et al., 2017).

Learning and memory is information that is encoded in the pattern of neuron firing after LTP and LTM processes, and during these processes the necessary biological mechanisms are attuned to temporal signals that are both internal and external. This review has considered the internal timing mechanisms in the first 24 h of a memory, yet this is only the starting point. The interactions between circadian, encoding and consolidating time codes in the first 24 h is perhaps the best-understood LTM research area and offers an opportunity to identify more optimal time patterns for learning in individuals and groups. This may, in turn, inform better social times for learning and work.

## Author contributions

PK: neuroscience. ME and JK: social time and sociology.

### Conflict of interest statement

PK is Chair of the Science Advisory Board of Download Learning, UK and on the Advisory Board of the charity Start School Later, U.S. The remaining authors declare that the research was conducted in the absence of any commercial or financial relationships that could be construed as a potential conflict of interest.
